# ﻿*Primulawolongensis* (Primulaceae), a new species of the primrose from Sichuan, China

**DOI:** 10.3897/phytokeys.218.91161

**Published:** 2023-01-10

**Authors:** Xiong Li, Yue-Hong Cheng, Hong-Qiang Lin, Cheng Chen, Xin-Fen Gao, Heng-Ning Deng, Feng Yu, Plenković-Moraj Anđelka, Wen-Bin Ju, Bo Xu

**Affiliations:** 1 China-Croatia “Belt and Road” Joint Laboratory on Biodiversity and Ecosystem Services, Key Laboratory of Mountain Ecological Restoration and Bioresource Utilization & Ecological Restoration Biodiversity Conservation, Chengdu Institute of Biology, Chinese Academy of Sciences, Chengdu 610041, Sichuan, China Chengdu Institute of Biology, Chinese Academy of Sciences Chengdu China; 2 University of Chinese Academy of Sciences, Beijing 100049, China University of Chinese Academy of Sciences Beijing China; 3 Sichuan Wolong National Natural Reserve Administration Bureau, Wenchuan 623006, Sichuan, China Sichuan Wolong National Natural Reserve Administration Bureau Wenchuan China; 4 Faculty of Science, University of Zagreb, Zagreb 10000, Croatia University of Zagreb Zagreb Croatia; 5 Key Laboratory of Bio-Resources and Eco-Environment of Ministry of Education, College of Life Sciences, Sichuan University, Chengdu 610065, Sichuan, China Sichuan University Chengdu China

**Keywords:** Hengduan Mountains, morphological characters, phylogenetic analysis, *Primula* sect. *Petiolares*, taxonomy

## Abstract

This paper describes and illustrates a new species of Primulaceae, *Primulawolongensis***sp. nov.** from Wolong National Nature Reserve in Sichuan Province, China. It is very rare and currently only known from its type locality. The new species belongs to subsection Chartacea of the section Petiolares on account of lacking bud scales at flowering, being efarinose and having distinct petiolate leaves with more or less rounded lamina. The new species can be differentiated from other members of the subsection by leaf blade margin dentate, and leaf veins which are not raised, scape shorter than or equal to pedicels, yellow flowers and location of stamens of the corolla tube at thrum flower. Molecular phylogenetic analysis based on nuclear ribosome internal transcribed spacer (nrITS) demonstrated that *P.wolongensis* was sister to subgen. Auriculastrum. *Primulawolongensis* is currently known from a single location in Wolong Town, and its conservation status is assessed as Data Deficient (DD).

## ﻿Introduction

*Primula* L. is the most widespread genus in Primulaceae. It contains about 500 species mainly distributed throughout the moister and cooler regions of the northern hemisphere (Hu 1990; Hu and Kelso 1996; APG 2016). The modern distribution center of the genus is the great mountain chain of the East Himalayan and Hengduan Mountains (Hu 1994; Hu and Kelso 1996). More than 300 species were found in this region, accounting for 78% of the total number of species (Richards 2003). The establishment of *Primulapetiolaris* Wall. and its allies as representing a distinct section of the genus was the work of Pax (1889). The sect. Petiolares has nearly 60 species, mainly distributed in the Himalayan range and the alpine regions of Western China (Hu 1994; Hu and Kelso 1996; Richards 2003). Its members are easily recognized by the capsule globose, included within the calyx-tube lacking valves and with an apical membrane crumbling at maturity (Hu and Kelso 1996). The sect. Petiolares was divided into 5 subsections according to bud-scales conspicuous and persisting at flowering time, length of scapes at flowering, and whether there is a honeycomb-reticulate below the blade, namely subsect. Chartacea (Balf.f.) Smith & Forrest, subsect. Davidii (Balf.f.) Craib, subsect. Griffithii Smith & Forrest, subsect. Petiolaris-Sonchifoli Smith & Forrest and subsect. Tongolensis Smith & Forrest (Smith and Fletcher 1944). In recent years, many new species have been reported in this section (Hu and Geng 2003; Li and Hu 2009; Rankin 2010; Hu and Hao 2011; Xu et al. 2014, 2015, 2016; Ju et al. 2018; Yuan et al. 2018) and it’s likely more undescribed taxon will be discovered.

In May 2021, an unusual population of *Primula* was discovered in moss-covered crevices of wet cliffs above the tree-line, in Sichuan Province. After consulting relevant literature (Smith and Fletcher 1944; Hu 1986; Hu 1990; Fang 1994, 2003; Hu and Kelso 1996; Wu 1999; Richards 2003) and herbarium specimens (BM, CDBI, E, FI, HNWP, IBSC, K, KUN, P, PE, US, and WU), we concluded that it is an undescribed taxon, belonging to Primulasect.Petiolares in morphology. Based on the morphological data of fresh materials and herbarium specimens, we describe this new species as follows.

## ﻿Materials and methods

### ﻿Morphological analyses

Morphological description and measurements of *Primulawolongensis* were based on living plants and dried herbarium specimens. The taxonomic description follows the terminology used by Beentje (2012). Voucher specimens and additional silica-gel dried leaves are stored at CDBI Herbarium (herbarium follows Thiers 2021).

### ﻿DNA extraction, amplification and sequencing

Except for the newly generated sequence of *Primulawolongensis* in this study, the sequences of the remaining 62 samples representing nine subgenera of *Primula* and two outgroups (*Androsaceintegra* and *A.paxiana*) in molecular phylogenetic analysis were retrieved from GenBank. Total DNA was extracted exclusively from silica-gel dried leaves via a Plant DNA Isolation Kit (Cat.No.DE-06111). We used the same primers as Xu et al. (2012) to amplify the nuclear ribosome internal transcribed spacer (nrITS) through polymerase chain reaction (PCR). All DNA samples were sent to TSINGKE Biotech Co. Ltd (Chengdu, China) for sequencing and then deposited to GenBank under the accession number OP901719.

### ﻿Phylogenetic analyses

All sequences were processed with Sequencher v4.1.4 (Gene Codes, Ann Arbor, Michigan, USA) and aligned using MAFFT v7.475 (Katoh and Standley 2013) with default parameters. We performed Maximum likelihood (ML) analysis based on nrITS dataset using IQ-TREE v1.4.2 (Nguyen et al. 2014) with branch support estimated by 2,000 replicates of ultrafast bootstrapping algorithm (UFboot) (Minh et al. 2013).

## ﻿Results

**Figure 1.**
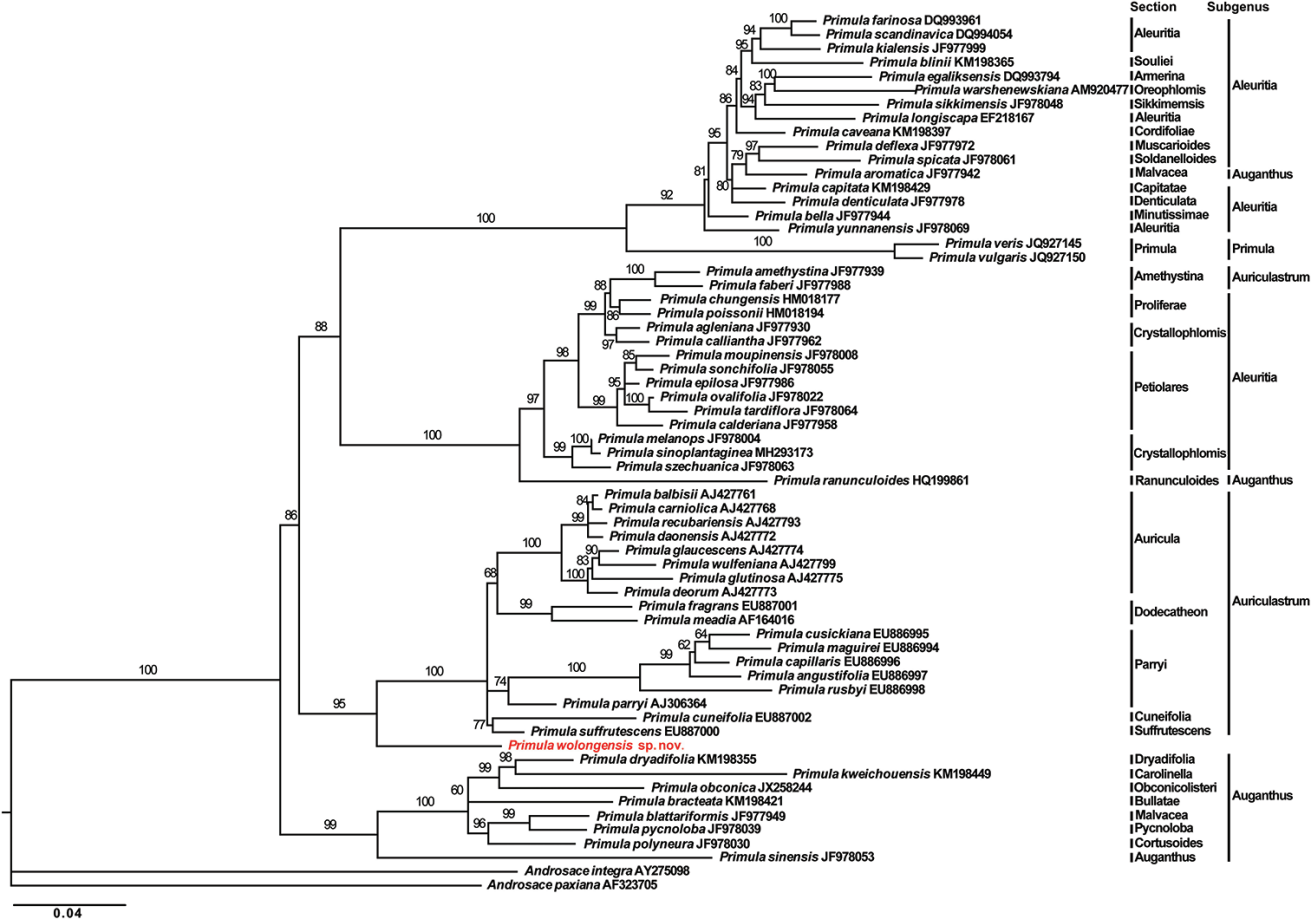
Maximum likelihood tree of *Primula* from phylogenetic analysis of nrITS sequence data.

The molecular phylogenetic tree showed that *Primulawolongensis* was sister to subgen. Auriculastrum (ML = 95, Fig. 1) which included sect. Auricula Duby, sect. Cuneifolia Balf.f., sect. Dodecatheon (L.) A.R.Mast & Reveal, sect. Parryi W.W.Sm. ex Wendelbo and sect. Suffrutescens A.J.Richards. Apart from the sect. Amethystina, which occurs in the Himalayan-Transverse Mountains, species of all other sections of this subgenus are found in Europe, North America, and northern Japan through to British Columbia. The position of *P.wolongensi*s in the genus *Primula* is clearly divergent between morphological and molecular evidence, and more evidence is needed to understand the evolutionary history of the species.

### ﻿Taxonomy

#### ﻿ Primula wolongensis

Taxon classificationPlantaeEricalesPrimulaceae

W.B.Ju, Bo Xu & X.F.Gao sp. nov.

0C0E648E-E4E9-5D7C-84F7-0C3006978959

urn:lsid:ipni.org:names:77311678-1

##### Diagnosis.

Amongst the Chinese members of subsect. Chartacea, the new species is easily recognized by the following combination of characters: leaf blade margin dentate, leaf veins which are not raised, scape which is shorter or equal with pedicel, corolla yellow and location of stamens of the corolla tube at thrum flower. The new species is morphologically similar to *P.arunachalensis* Basak & Maiti and *P.fenghwaiana* C.M.Hu & G.Hao, but can be easily distinguished from *P.arunachalensis* by its shorter rootstocks, petioles 3–5× as long as leaf blade (versus 1–2× as long as leaf blade), reticulation of veins obscure on both surfaces (versus veins slightly impressed adaxially and conspicuous abaxially), corolla lobes apex emarginate (versus corolla lobes margin denticulate to lacerate), heterostylous (versus homostylous). Compared with *P.fenghwaiana*, the difference of the new species is the petioles 3–5× as long as the leaf blade (versus ca.2/3 the length of the leaf blade), leaf blade base cordate (versus base broadly cuneate to almost rounded), leaf blade margin irregular dentate (versus margin remotely denticulate), scapes and pedicels sparsely short-stalked glandular (versus densely covered with minute glandular hairs), corollas yellow (versus pink to white), the position of stamens at thrum flower tube on the middle (versus on the apex).

##### Type.

**Figure 2.**
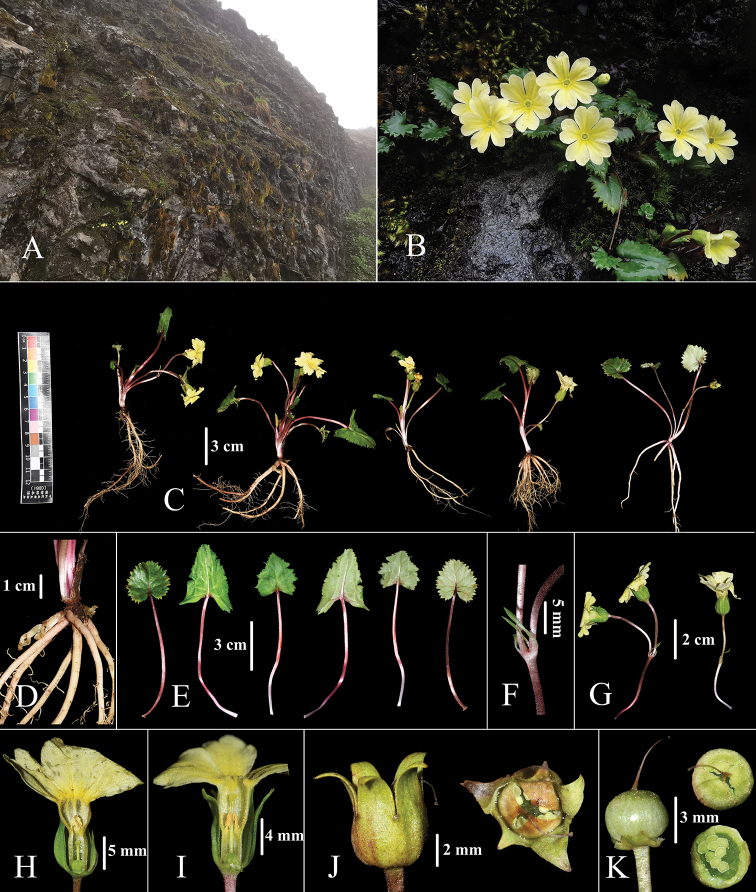
*Primulawolongensis* sp. nov. **A** habitat **B** habit **C** whole plant **D** roots and second-year buds **E** leaves **F** bracts **G** scape **H** thrum flower **I** pin flower **J** calyx in fruit **K** capsule.

**Figure 3.**
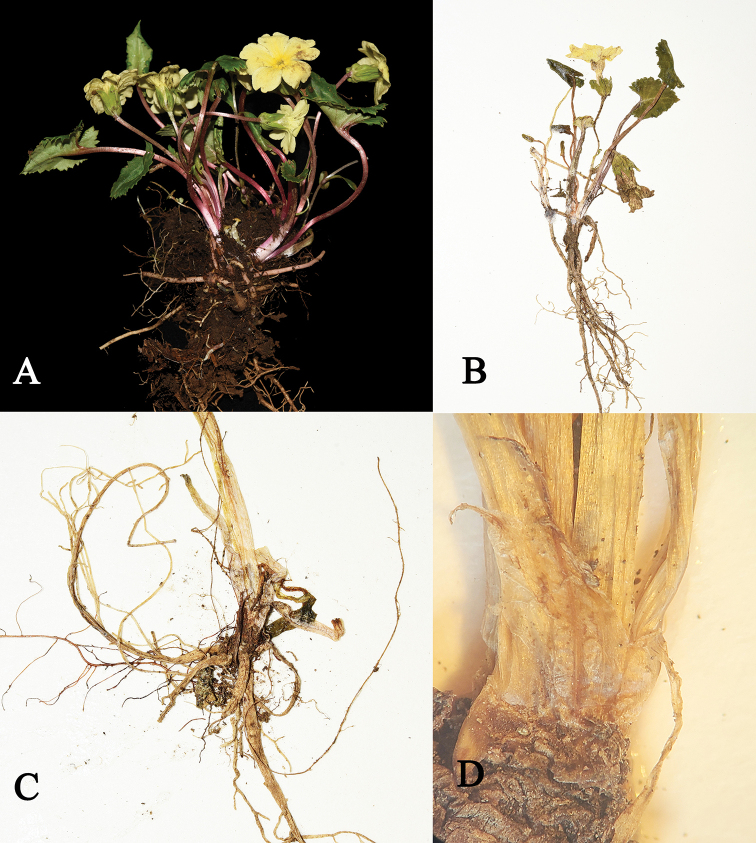
*Primulawolongensis* sp. nov. **A** fresh plants **B** pressed specimen **C** decayed persistent petiole **D** remaining scales.

**Figure 4.**
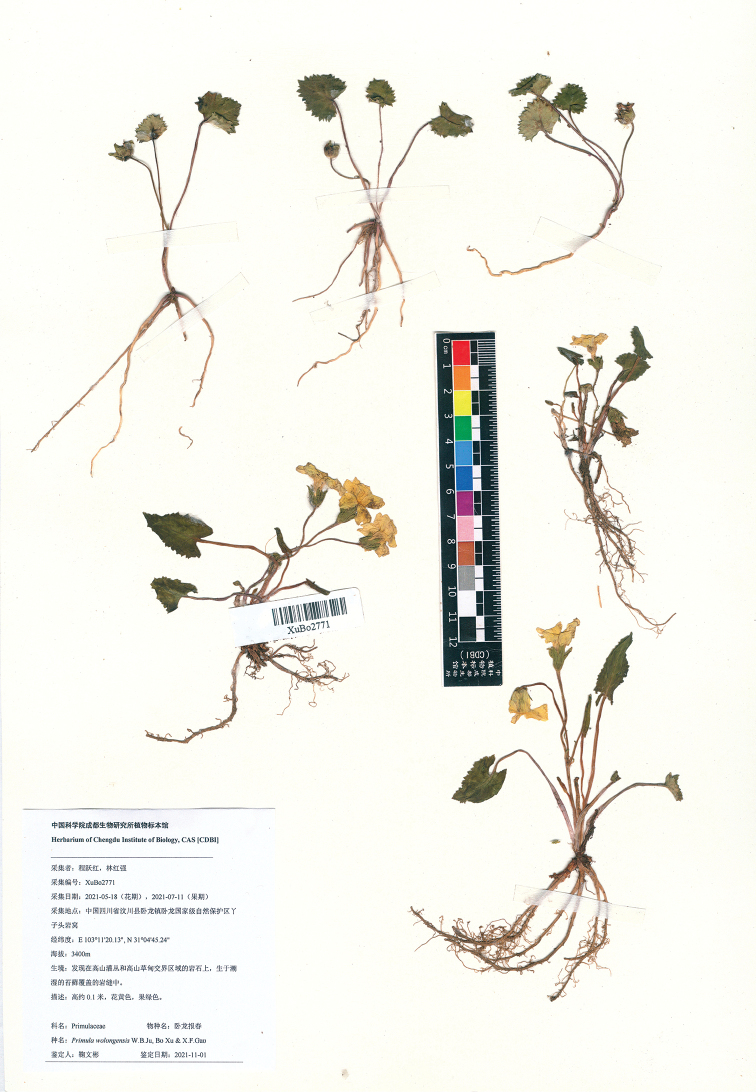
Type sheet of *Primulawolongensis*.

China. Sichuan: Wenchuan City, Wolong National Nature Reserve, growing in moist rock crevices covered with moss. 31°04'N, 103°11'E, elevation ca. 3400 m, 18 May 2021 (fl./fr.), *Y.H. Cheng & H.Q. Lin* XuBo2771 (holotype CDBI!; isotypes KUN!, PE!). (Figs 2–4)

##### Description.

A perennial plant, efarinose, having reddish-brown basal bud scales, second-year buds at the base and free from bud scales at flowering time, rootstock extremely short. ***Roots*** numerous, fibrous. ***Leaves*** forming a loose rosette; young leaf blade often fold, widely ovate to suborbicular at maturity, 1.0–3.5 × 1–2.5 cm, broadly obtuse to rounded at apex, with a cordate base, margin irregular dentate, glabrous on both surfaces, glaucous below, firm papery when dry, lateral veins 3–5 pairs, slightly impressed adaxially, reticulation of veins obscure on both surfaces; petioles 3.0–8.5 cm long, sparsely short-stalked glandular, reddish brown. ***Scape*** 1, sparsely short-stalked glandular, 1.8–4.5 cm tall, non-elongating at fruiting time; umbel with 1–3 flowers; bracts linear to linear-lanceolate, 0.7–1.5 cm long; pedicels 1.5–4.5 cm long, densely short-stalked glandular, not extended in fruit. ***Flowers*** heterostylous. ***Calyx*** green, campanulate, 6–11 mm long, slightly enlarged, poculiform in fruit, short-stalked glandular, parted slightly below middle to 2/3; lobes ovate-lanceolate, margin entire, apex acute. ***Corolla*** yellow with short-stalked glandular, annulate; limb 16–25 mm across, funnelform; lobes spreading, 7–12 × 5–10 mm, broadly obovate, emarginate. ***Thrum flower***: corolla tubes 8–12 mm in length, 3–4 mm in diameter, slightly longer than calyx, widely ampliated above insertion of stamens; stamens inserted slightly above the middle of corolla tube; style ca. 4 mm. ***Pin flower***: corolla tubes 7–10 mm in length, ca. 3 mm in diameter, nearly equal to calyx, widely ampliated above insertion of stamens; stamens in the middle of corolla tube, style ca. 2/3 as long as tube. ***Capsule*** globose, included in calyx, disintegrating at maturity.

##### Phenology.

Flowering May-June, fruiting May-August.

##### Etymology.

The specific epithet refers to the type locality, Wolong National Nature Reserve.

##### Vernacular name.

A Chinese name, wo long bao chun (卧龙报春), is suggested here.

##### Distribution and habitat.

The species has so far only been found at its type locality in Wolong Town, Wenchuan county, Sichuan Province. It grows in the cracks of steep wet cliffs covered with moss above the tree-line.

##### Conservation status.

Data Deficient (DD). Currently, only one population with more than 100 individuals has been found in the type locality. According to the guidelines for using the IUCN Red List categories and criteria (IUCN Standards and Petitions Subcommittee 2022), the conservation status of the new species is ‘Data Deficient (DD)’. Further explorations in the adjacent mountainous tracts are necessary for an adequate assessment.

##### Discussion.

Following Hu’s (1990) taxonomic treatment of this genus, this new species belongs to PrimulasectionPetiolares on account of globose capsule included within the calyx-tube that does not open by valves but apically crumbling at membrane apex at maturity (Figs 2–4).

Further morphological analysis shows that the new species is allied with subsect. Chartacea by having glabrous and efarinose plants, at flowering time devoid of basal bud-scales, more or less rounded blades and slender petioles.

Including the newly described here, there are nine species reported so far for this subsection (Smith and Fletcher 1944; Hu 1990; Basak and Maiti 2000; Rankin 2010; Hu and Hao 2011; Xu et al. 2015; Wei et al. 2022). Amongst the Chinese members of subsect. Chartacea, the new species, is morphologically most similar to *P.arunachalensis* and *P.fenghwaiana* in its bud-scales lacking at anthesis, slender petioles, rounded blades, and scape shorter than or equal to pedicels, but can be recognized by almost absent rhizomes, morphological features of leaves, inflorescences, and flowers. Further morphological comparisons among *P.wolongensis*, *P.arunachalensis* and *P.fenghwaiana* are shown in Table 1.

**Table 1.** Comparison of morphological characters among *Primulawolongensis*, *P.arunachalensis* and *P.fenghwaiana*.

Characters	*P.wolongensis*	*P.arunachalensis*	*P.fenghwaiana*
Habitat	moss-covered crevices of wet cliffs	on hill slope	secondary evergreen broad-leaved forests
Rootstock	less than 0.3 cm long	ca. 2 cm long	1–2 cm long
Petioles	3–5× as long as leaf blade	1–2× as long as leaf blade	ca.2/3 the length of the leaf blade
Leaf blade	widely ovate to suborbicular	orbicular	broadly elliptic to broadly obovate
	the veins are not raised on both sides	the veins are raised on both sides	the veins are raised on both sides
	base cordate	base cordate to truncate	base broadly cuneate to almost rounded
	margin dentate	margin irregularly and shallowly dentate to denticulate	margin remotely denticulate
Scapes	1.8–4.5 cm, sparsely short-stalked glandular	ca. 1.5 cm, glabrous	0.8–1.2 cm, densely covered with minute glandular hairs
	as long as pedicel	longer than pedicel	shorter than pedicel
Calyx	campanulate, poculiform in fruit	campanulate	narrowly campanulate
Corolla	yellow, heterostylous, annulate	pale yellow, homostylous, exannulate	pink to white, heterostylous, annulate
	lobes emarginate	lobules dentate or lacerate	lobes bilobed, lobules entire or toothed
Stamens	inserted on the middle of the corolla tube at thrum flower	inserted on the apex of the corolla tube at thrum flower	inserted on the apex of the corolla tube at thrum flower

## Supplementary Material

XML Treatment for Primula wolongensis
